# Utilizing Bladder Diaries to Prevent Unnecessary Treatment in Patients With Storage Dysfunction: A Retrospective Study

**DOI:** 10.7759/cureus.68437

**Published:** 2024-09-02

**Authors:** Kanya Kaga, Kosuke Mikami, Mayuko Kaga

**Affiliations:** 1 Department of Urology, Chiba Prefectural Sawara Hospital, Katori, JPN; 2 Department of Urology, Chiba University Hospital, Chiba, JPN; 3 Department of Urology, Mihama Narita Clinic, Narita, JPN

**Keywords:** iciq-ui sf, storage symptoms, oabss, overactive bladder symptom score, bladder diary

## Abstract

Introduction

A frequency volume chart (FVC) or bladder diary (BD) is used to diagnose lower urinary tract symptoms and to determine the effectiveness of treatment. In outpatient practice, patients who use an FVC or BD may experience improvement in storage symptoms and not desire further treatment. The aim of this study was to determine the characteristics of patients who did not desire treatment after BD recording and to assess the changes in storage symptoms after BD recording.

Methods

This was a retrospective study. Patients who completed a three-day BD record were included. The patients were divided into two groups: those whose symptoms improved after using a BD and no longer desired treatment, and those who desired treatment. We compared endpoints including patient background, BD, Overactive Bladder Symptom Score (OABSS), and International Consultation on Incontinence Questionnaire-Urinary Incontinence Short Form (ICIQ-UI SF) score.

Results

We recruited 79 patients. Four patients were excluded (two were minors and two due to cognitive impairment). Thus, 75 patients were included in the analysis. Of these, 27 (36.0%) did not desire treatment for storage symptoms after BD recording. Compared with the group of patients who desired treatment, those who did not desire treatment had significantly lower daytime and nighttime frequency and number of leaks recorded in their BD, and there were fewer patients with nocturia and habitual caffeine consumption. Baseline OABSS and ICIQ-UI SF scores were lower and there were no significant changes in storage symptoms after BD recording. The cut-off value for the baseline total score of OABSS that did not desire treatment for storage symptoms after BD recording was 6 points.

Conclusion

In this study, 36.0% of patients no longer desired treatment for storage symptoms after using a BD. These patients initially exhibited a normal daily urinary frequency and mild storage symptoms. These findings suggest that among patients presenting to the outpatient clinic with storage symptoms, those with mild symptom severity and a total OABSS of 6 points or less may be able to avoid unnecessary treatment through BD recording.

## Introduction

A frequency volume chart (FVC) or bladder diary (BD) is the recommended method of assessment for lower urinary tract symptoms [1,2]. An FVC is a method of recording the time of each micturition together with the volume voided for at least 24 hours, whereas a BD includes additional information such as the fluid intake, pad usage, incontinence episodes, the degree of incontinence, and the circumstances at the time of the leakage [3]. An FVC or BD is often introduced early, as it provides important information for the diagnosis of polyuria, nocturnal polyuria, and psychogenic urinary dysfunction [4]. Among patients with storage symptoms, symptoms may improve, and treatment may no longer be required while being assessed using these methods.

This study focused on patients who did not desire treatment for their storage symptoms after completion of BD recording. The aim of this study was to compare differences in backgrounds between patients who desired treatment and those who did not after BD recording and to assess changes in storage symptoms after BD recording.

## Materials and methods

This was a retrospective study. The Ethical Review Board of our institution approved the study, and consent to participate was obtained in an opt-out format.

Patients who presented to the outpatient clinic with storage symptoms and completed a three-day BD record were included. Patients who were minors, pregnant or possibly pregnant, and who had a history of urinary tract infection, urinary tract stones, urethral disease, or pelvic surgery within 180 days prior to BD recording were excluded.

Endpoints included patient background, such as age, sex, body mass index, and disorders causing storage symptoms; daytime and nighttime frequency; the numbers of urgency incontinence and leaks episodes; voided volume; the amount of leaks; nocturnal polyuria index (NPi); hours of undisturbed sleep; urine volume per body weight; daily and nighttime fluid intake; caffeine and alcohol habituation; Overactive Bladder Symptom Score (OABSS); and International Consultation on Incontinence Questionnaire-Urinary Incontinence Short Form (ICIQ-UI SF) score. OABSS and ICIQ-UI SF were assessed before and after BD recording.

OABSS is a four-item questionnaire that quantifies storage symptoms in a single score. The maximal score is defined as 2, 3, 5, and 5 for daytime frequency, nighttime frequency, urgency, and urgency incontinence, respectively. The total score ranges from 0 to 15, with higher scores indicating more severe storage symptoms [5,6]. ICIQ-UI SF is a questionnaire to assess urinary incontinence. The maximal score is defined as 5, 6, and 10 for frequency, severity, and impact on the quality of life of urinary incontinence. The total score ranges from 0 to 21, with higher scores indicating more severe urinary incontinence [7]. NPi is calculated using the formula (nighttime urine volume/24 h urine volume) × 100% [3].

After completion of BD recording, patients were interviewed about their desire for treatment for storage symptoms and divided into two groups: patients who no longer desired treatment (Group A), and those who desired treatment (Group B), and each endpoint was compared.

Statistical analysis

The results are shown as the mean and standard deviation. Statistical analysis was performed using JMP software, version 14.2.0 (SAS Institute Inc., Cary, N.C., USA). Wilcoxon rank-sum test was performed to assess statistical differences in patient background and BD items, baseline OABSS, and ICIQ-SF scores. Wilcoxon signed-rank test was performed to examine the significance of the changes in OABSS and ICIQ-SF scores between the baseline and after BD recording in each of the two groups. The receiver operating characteristic (ROC) curve was calculated with Groups A and B as objective variables, and the baseline OABSS total score as the explanatory variable. P-value < 0.05 was considered significant.

## Results

Seventy-nine patients presented to the outpatient clinic with storage symptoms and completed the three-day BD recording. Of these, four patients were excluded (two were minors and two due to cognitive impairment). Seventy-five patients were included in the analysis, of whom 27 (36.0%) had symptoms that improved after BD recording and did not desire treatment for storage symptoms. The patient background of the two groups is shown in Table 1.

**Table 1:** Baseline characteristics of patients with storage symptoms who participated in this study Group A: patients who no longer desired treatment; Group B: patients who desired treatment Overactive bladder was defined as a total overactive bladder symptom score of at least 3 points and an urgency score of at least 2 points. Polyuria was defined as 24-hour urinary output that exceeds 40 mL/kg body weight. Nocturnal polyuria was defined as urine volume during the nighttime that is more than 33% of the total 24-hour urine output. Nocturia was defined as waking up more than twice to urinate during the main sleep period. Wilcoxon rank-sum test was performed to assess statistical differences in patient background.

Characteristics	Group A (N = 27)	Group B (N = 48)	P-value
Male/female	14/13	25/23	1.00
Age, years	69.4 ± 13.3	71.4 ± 11.4	0.52
Body mass index, kg/m^2^	23.1 ± 3.2	23.4 ± 4.5	0.70
Prostate volume, mL	30.0 ± 14.3	36.4 ± 22.1	0.30
Overactive bladder	9	24	0.12
Polyuria	1	5	0.29
Nocturnal polyuria	16	22	0.19
Nocturia	6	23	0.02
Psychogenic urinary dysfunction	0	2	0.41
Stress urinary incontinence	0	2	0.41

Although there were no significant differences in patient backgrounds, such as sex or age, the percentage of patients in Group A who woke up to go to the bathroom two or more times during the nighttime was significantly lower than in Group B. BD results for the two groups are shown in Table 2.

**Table 2:** Bladder diary in the two groups Group A: patients who no longer desired treatment; Group B: patients who desired treatment Wilcoxon rank-sum test was performed to assess statistical differences in the various bladder diary items.

	Group A (N = 27)	Group B (N = 48)	P-value
Daytime frequency	6.1 ± 1.5	8.5 ± 2.6	<0.01
Nighttime frequency	1.2 ± 0.7	2.2 ± 1.5	<0.01
Number of urgency episodes/24 hour	0.6 ± 1.0	1.5 ± 3.0	0.09
Number of leaks/24 hour	0.1 ± 0.3	1.1 ± 2.3	0.01
Total voided volume/24 hour, mL	1491.1 ± 593.0	1611.7 ± 658.1	0.43
Total voided volume/nighttime, mL	591.5 ± 236.8	603.7 ± 282.4	0.87
Nocturnal polyuria index, %	38.8 ± 0.1	37.9 ± 0.1	0.72
Amount of leaks/24 hour, mL	36.5 ± 166.6	60.7 ± 174.9	0.60
Maximum voided volume, mL	367.2 ± 150.7	312.3 ± 160.5	0.17
Mean voided volume/24 hour, mL	200.7 ± 68.0	161.7 ± 82.5	0.06
First nighttime voided volume, mL	280.3 ± 96.6	228.7 ± 145.7	0.12
Hours of undisturbed sleep, hour	4.8 ± 2.1	3.6 ± 2.2	0.06
Urine/kg body weight, mL/kg	25.0 ± 8.7	27.8 ± 11.4	0.25
Fluid intake/24 hour, mL	1642.9 ± 715.3	1316.9 ± 562.1	0.13
Fluid intake/nighttime, mL	101.2 ± 138.1	101.2 ± 165.3	0.25
Regular caffeine consumption	13	35	0.03
Regular alcohol consumption	5	7	0.44

Daytime and nighttime frequency were significantly lower in Group A and the original storage symptoms were milder. On the other hand, there were no significant differences in the voided volume or the amount of leaks. Although there was no significant difference in daily or nighttime fluid intake, a smaller percentage of Group A habitually consumed caffeine compared with Group B. The results of the OABSS and ICIQ-UI SF before BD recording for the two groups are shown in Table 3.

**Table 3:** OABSS and ICIQ-UI SF at baseline Group A: patients who no longer desired treatment; Group B: patients who desired treatment; OABSS: overactive bladder symptom score; ICIQ-UI SF: International Consultation on Incontinence Questionnaire-Urinary Incontinence Short Form The total OABSS score ranges from 0 to 15, with higher scores indicating more severe storage symptoms. The total ICIQ-UI SF score ranges from 0 to 21, with higher scores indicating more severe urinary incontinence. Wilcoxon rank-sum test was performed to assess statistical differences in baseline OABSS and ICIQ-UI SF scores.

	Group A (N = 27)	Group B (N = 48)	P-value
OABSS			
Total score, points	4.0 ± 2.3	7.0 ± 3.9	<0.01
Frequency score, points	0.5 ± 0.5	0.9 ± 0.5	<0.01
Nocturia score, points	1.8 ± 0.9	2.3 ± 1.0	<0.01
Urgency score, points	1.1 ± 1.3	2.1 ± 1.9	<0.01
Urgency incontinence score, points	0.5 ± 0.8	1.6 ± 1.9	<0.01
ICIQ-UI SF			
Total score	2.2 ± 2.3	6.7 ± 5.9	<0.01
Frequency of leaks score, points	0.8 ± 0.9	2.1 ± 2.0	<0.01
Amount of leaks score, points	0.9 ± 1.0	1.9 ± 1.7	<0.01
Quality of life score, points	0.5 ± 0.9	2.7 ± 3.2	<0.01

**Figure 1:**
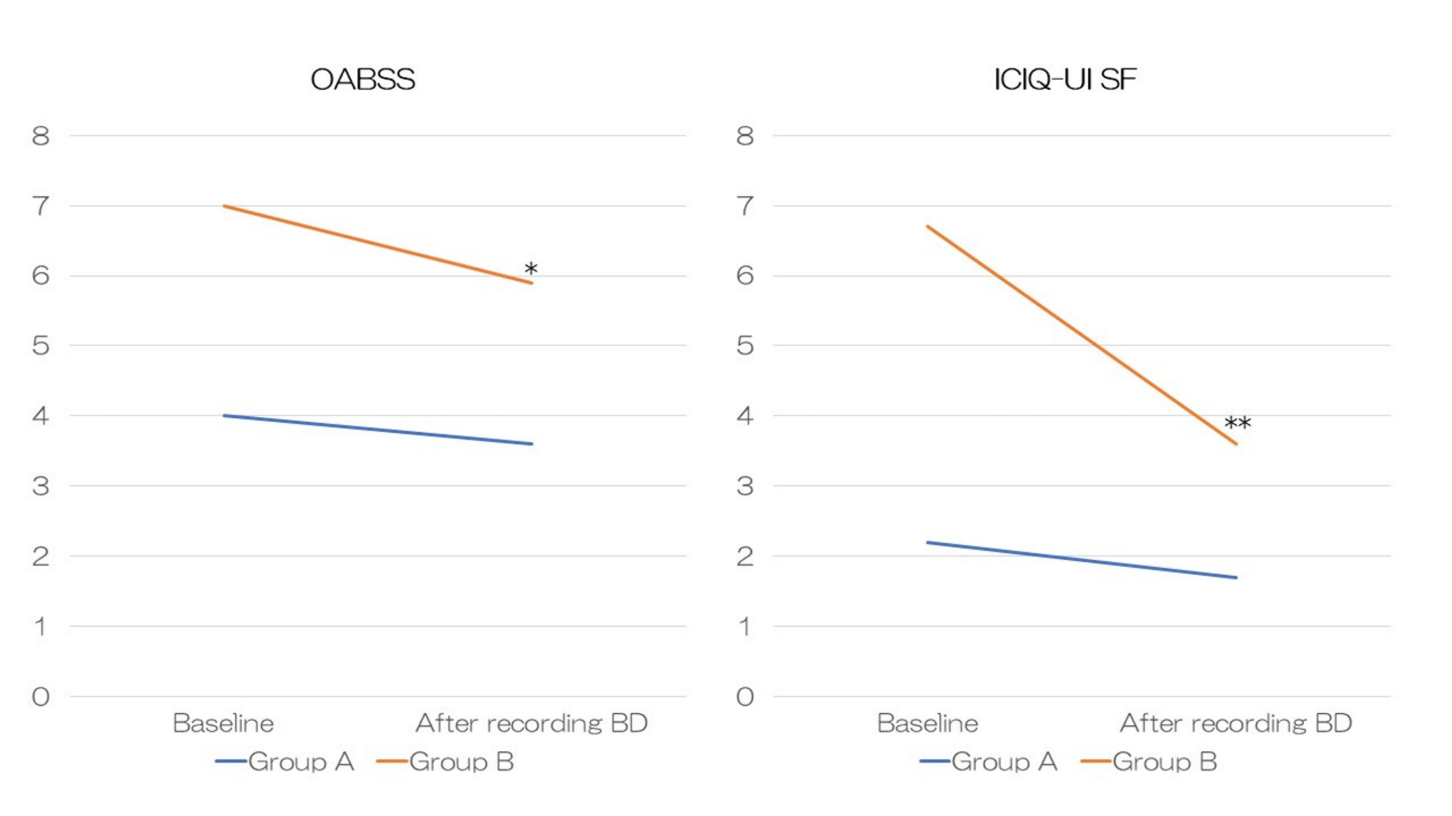
Changes in OABSS and ICIQ-UI SF after recording BD Group A: patients who no longer desired treatment; Group B: patients who desired treatment; OABSS: overactive bladder symptom score; ICIQ-UI SF: International Consultation on Incontinence Questionnaire-Urinary Incontinence Short Form; BD: bladder diary Wilcoxon signed-rank test was performed to examine the significance of the changes in OABSS and ICIQ-UI SF scores between the baseline and after BD recording in each of the two groups. *P < 0.05, **P < 0.01 versus baseline

Both the baseline OABSS and ICIQ-UI SF total scores and subscores were significantly lower in Group A than in Group B. Changes in OABSS and ICIQ-UI SF after BD recording are shown in Figure 1.

**Figure 2:**
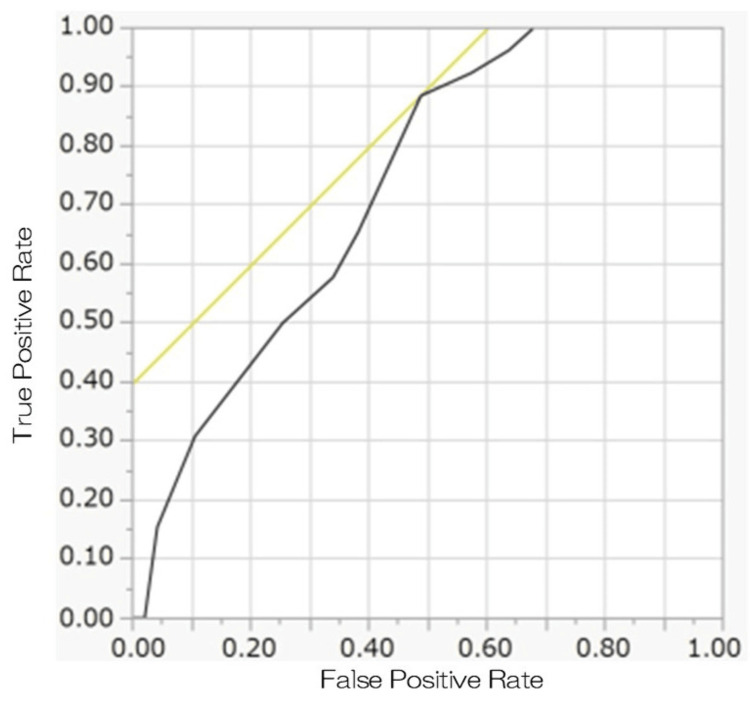
ROC curve generated from the baseline OABSS total score in the two groups The black curve: ROC curve; the yellow curve: the tangent line of ROC curve; ROC: receiver operating characteristic; OABSS: overactive bladder symptom score The area under the curve was 0.73.

Group A showed no significant changes in OABSS or ICIQ-UI SF after BD recording. On the other hand, Group B showed significant decreases in both OABSS and ICIQ-UI SF. The ROC curve generated from the baseline OABSS total score in the two groups is shown in Figure 2.

The area under the curve was 0.73. The ROC table output during ROC curve generation is shown in Table 4.

**Table 4:** ROC table output during ROC curve generation ROC: receiver operating characteristic; OABSS: overactive bladder symptom score. *: The cut-off value

The baseline OABSS total score	Probability	1-Specificity	Sensitivity	Sensitivity-(1-Specificity)	True positive	True negative	False positive	False negative
.	.	0.0000	0.0000	0.0000	0	48	0	27
1	0.6538	0.0417	0.1481	0.1065	4	46	2	23
2	0.5873	0.1250	0.3333	0.2083	9	42	6	18
3	0.5175	0.2708	0.5185	0.2477	14	35	13	13
4	0.4469	0.3542	0.5926	0.2384	16	31	17	11
5	0.3785	0.3958	0.6667	0.2708	18	29	19	9
6*	0.3145	0.5000	0.8889	0.3889	24	24	24	3
7	0.2569	0.5833	0.9259	0.3426	25	20	28	2
8	0.2067	0.6458	0.9630	0.3171	26	17	31	1
9	0.1641	0.6875	1.0000	0.3125	27	15	33	0
10	0.1289	0.7917	1.0000	0.2083	27	10	38	0
11	0.1003	0.8333	1.0000	0.1667	27	8	40	0
12	0.0775	0.8958	1.0000	0.1042	27	5	43	0
13	0.0595	0.9375	1.0000	0.0625	27	3	45	0
14	0.0455	0.9792	1.0000	0.0208	27	1	47	0
15	0.0347	1.0000	1.0000	0.0000	27	0	48	0

The cut-off value for the baseline total score of OABSS that did not desire treatment for storage symptoms after BD recording was 6 points.

## Discussion

FVC and BD provide important insights into lower urinary tract symptoms that cannot be determined through outpatient evaluation alone. However, their reliability is controversial because the patient is responsible for recording the data. As a previous study reported no difference in reliability between three-day and seven-day records, in this study, a three-day BD record was utilized [8].

To the best of our knowledge, there are no reports examining the background of patients whose symptoms improved after BD recording and who no longer desired treatment for storage symptoms. The total daytime and nighttime frequency in adults without storage symptoms has been reported to range from six to seven times a day [9-11]. OABSS scores correlate with the severity of OAB symptoms [12]. In previous studies, scores of 5 or less were classified as mild [13,14]. This suggests that Group A consisted of patients whose urinary frequency was comparable to that of asymptomatic adults and whose storage symptoms were mild. Furthermore, the cut-off value for the baseline OABSS total score in the two groups was 6 points (Table 4); thus, our findings suggest that advising patients with mild storage symptoms, especially those with a total OABSS score of 6 points or less, to use a BD helps avoid subsequent treatment. We speculate that patients with mild storage symptoms may have had normal urinary frequency and other symptoms; however, they misinterpreted their condition as storage symptoms. On the other hand, half of the patients in Group B had nocturia, suggesting that the bladder training effect may have significantly improved their storage symptoms. The bladder training effect is a phenomenon in which the use of FVC affects nighttime frequency [15]. Group B also had a higher proportion of patients who habitually consumed caffeine. Caffeine intake has been reported to play a role in storage symptoms and may have contributed to the perception that symptoms had not improved after BD recording [16].

In this study, no information was provided beforehand that could be related to changes in patients’ storage symptoms. It was confirmed through interviews conducted after BD recording that patients did not intentionally use behavioral therapies such as salt restriction or wearing compression stockings during the daytime [17,18]. However, a limitation of the study is that patients may have voluntarily used behavioral therapy during the study period, as OABSS and ICIQ-UI SF scores in Group B improved. On the other hand, there were no significant changes in OABSS or ICIQ-UI SF scores after BD recording in Group A. This may be explained by these patients originally having normal urinary frequency and mild storage symptoms, irrespective of the effects of voluntary behavioral therapy or bladder training.

## Conclusions

The present study assessed the characteristics of patients who no longer desired treatment for storage symptoms after BD recording. Of the patients who complained of storage symptoms and used BD, 36.0% were able to avoid further treatment. Those who did not desire treatment after using BD originally had the same urinary frequency as asymptomatic adults and had mild storage symptoms. BD recording may be useful to avoid unnecessary treatment in patients with mild storage symptoms, especially in those with a total OABSS score of 6 points or less.
